# Differences in mucilage properties and stomatal sensitivity of locally adapted *Zea mays* in relation with precipitation seasonality and vapour pressure deficit regime of their native environment

**DOI:** 10.1002/pld3.519

**Published:** 2023-08-17

**Authors:** Bernd J. Berauer, Asegidew Akale, Andreas H. Schweiger, Mathilde Knott, Dörte Diehl, Marc‐Philip Wolf, Ruairidh J. H. Sawers, Mutez A. Ahmed

**Affiliations:** ^1^ Institute of Landscape and Plant Ecology, Department of Plant Ecology University of Hohenheim Stuttgart Germany; ^2^ Root‐Soil Interaction, TUM School of Life Sciences Technical University of Munich Freising Germany; ^3^ Institute for Environmental Sciences, Group of Environmental and Soil Chemistry RPTU in Landau Landau Germany; ^4^ Department of Plant Science The Pennsylvania State University State College Pennsylvania USA

**Keywords:** breeding, climate change, drought, mucilage, photosynthesis, plant, root, stomata regulation, *Zea mays*

## Abstract

With ongoing climate change and the increase in extreme weather events, especially droughts, the challenge of maintaining food security is becoming ever greater. Locally adapted landraces of crops represent a valuable source of adaptation to stressful environments. In the light of future droughts—both by altered soil water supply and increasing atmospheric water demand (vapor pressure deficit [VPD])—plants need to improve their water efficiency. To do so, plants can enhance their access to soil water by improving rhizosphere hydraulic conductivity via the exudation of mucilage. Furthermore, plants can reduce transpirational water loss via stomatal regulation. Although the role of mucilage and stomata regulation on plant water management have been extensively studied, little is known about a possible coordination between root mucilage properties and stomatal sensitivity as well as abiotic drivers shaping the development of drought resistant trait suits within landraces. Mucilage properties and stomatal sensitivity of eight Mexican landraces of *Zea mays* in contrast with one inbred line were first quantified under controlled conditions and second related to water demand and supply at their respective site of origin. Mucilage physical properties—namely, viscosity, contact angle, and surface tension—differed between the investigated maize varieties. We found strong influences of precipitation seasonality, thus plant water availability, on mucilage production (*R* ^2^ = .88, *p* < .01) and mucilage viscosity (*R* ^2^ = .93, *p* < .01). Further, stomatal sensitivity to increased atmospheric water demand was related to mucilage viscosity and contact angle, both of which are crucial in determining mucilage's water repellent, thus maladaptive, behavior upon soil drying. The identification of landraces with pre‐adapted suitable trait sets with regard to drought resistance is of utmost importance, for example, trait combinations such as exhibited in one of the here investigated landraces. Our results suggest a strong environmental selective force of seasonality in plant water availability on mucilage properties as well as regulatory stomatal effects to avoid mucilage's maladaptive potential upon drying and likely delay critical levels of hydraulic dysfunction. By this, landraces from highly seasonal climates may exhibit beneficial mucilage and stomatal traits to prolong plant functioning under edaphic drought. These findings may help breeders to efficiently screen for local landraces with pre‐adaptations to drought to ultimately increase crop yield resistance under future climatic variability.

## 1 INTRODUCTION

Drought events, which are increasing in frequency and severity with ongoing climate change (Dai, 2013; Masson‐Delmotte et al., 2021), drastically reduce crop yield, fueling the risk of food insecurity (Lesk et al., 2016; Vogel et al., 2019). To counteract the increasing risk of food insecurity, agricultural crops need to provide constant and sufficient yield even under future fluctuating climatic conditions. Landraces are a valuable source of genetic variation and unique adaptations to local environmental conditions (Lopes et al., 2015; Toulotte et al., 2022). They are genetically diverse dynamic populations of historically cultivated plants, which are locally adapted and associated with traditional farming practices (Camacho Villa et al., 2005). Identifying and quantifying trait suites involved in enhancing landrace water management and abiotic conditions shaping the development of these trait suits offer a promising avenue to leverage natural diversity (Toulotte et al., 2022). This understanding will help breeding programs aim to increase crop yield resistance and resilience to drought stress. Research on linking plant below‐ and aboveground morphological traits has already been conducted (Ávila‐Lovera et al., 2022; Mommer & Weemstra, 2012), yet little is known on the coordination between root mucilage characteristics (production as well as physical properties) and stomatal sensitivity, which reflect two different mechanisms for plants to improve their water management. Thus, initiating investigations is necessary to foster research in the coordination between those below‐ and aboveground plant ecophysiologic properties related to drought resistance and environmental factors driving their development.

Water is transported through the soil‐plant‐atmosphere continuum following a water potential gradient, respectively, a suction‐tension gradient, from the leaf‐atmosphere interface to the root‐soil interface (Grossiord et al., 2020; Mencuccini et al., 2019). Atmospheric water demand creates tension via transpiration at the leaf surface. This tension propagates through the plant all the way to the root tips. As a result, roots extract water from the soil to sustain transpirational demand. Thus, the boundaries at the root‐soil and leaf‐atmosphere interface are the sites within a plant to access (at the roots) and lose water (at the leaf surface via stomata), hence are crucial to plant drought resistance.

Soil moisture availability at the root‐soil boundary is directly influenced by precipitation and the hydraulic conductivity of the rhizosphere determines root water uptake (Passioura, 1980). Climate change alters mean and seasonality of precipitation patterns, directly affecting soil water balance and water availability for plants (Porporato et al., 2004; Vereecken et al., 2022). Plants have the ability to influence hydraulic conductivity at the root‐soil interface to improve root water uptake via root exudates (Ahmed, Passioura, & Carminati, 2018; Walker et al., 2003). Plants exude around 25% of their total photosynthetic product into the rhizosphere, nearly half of which is in the form of mucilage (Chaboud, 1983; Walker et al., 2003). Mucilage promotes plant growth by enhancing physio‐chemical properties within the root‐soil boundary layer, such as soil aggregation, lubricating root surfaces to improve soil penetration, and trapping and solubilizing nutrients (Ahmed et al., 2014; Bengough & McKenzie, 1997; Carminati et al., 2010). Mucilage can absorb huge amounts of water, changing the physical properties of the rhizosphere, keeping it moist and conductive even under drying soil (Ahmed et al., 2014; Carminati et al., 2010; Naveed et al., 2019). The high viscosity of mucilage at the root surface is linked to a reduction in surface tension, which helps to maintain root‐soil conductivity during soil drying (Benard et al., 2019; Naveed et al., 2019). However, depending on the environmental conditions such as the soil solution (Knott et al., 2022), mucilage can turn hydrophobic after drying, forming a water repellent region around the roots (Ahmed et al., 2016; Carminati et al., 2010). The positive effects of moist mucilage on plant water and nutrient uptake need to be balanced with the negative effects of dry mucilage and the exhibited hysteresis during the drying‐rewetting cycles within the rhizosphere (Carminati, 2012; Naveed et al., 2019). To circumvent the maladaptive effects of dry mucilage, regulatory mechanisms to avoid excessive transpirational water loss, for example, via stomatal sensitivity, might play an important role.

At the leaf‐atmosphere boundary, vapor pressure deficit (VPD; the difference between actual and saturated atmospheric water vapor) determines atmospheric water demand and influences stomata controlled plant transpiration (Yuan et al., 2019). As a result of climate change, VPD increased globally in recent years (Yuan et al., 2019; Zhang et al., 2015) and is predicted to further increase over the next century (Park Williams et al., 2013), already affecting terrestrial ecosystem functioning and evapotranspiration (see Yuan et al., 2019). Plants regulate their transpirational water loss by altering stomatal opening (Ache et al., 2010), while simultaneously controlling CO_2_ intake for photosynthesis (Brodribb & Holbrook, 2003; Yang et al., 2020). Stomatal opening is regulated by hydro‐active and hydro‐passive feedback loops (Buckley, 2019), partly governed by the VPD at the leaf surface (Running, 1976). Stomata regulation as a response to atmospheric drought, by increasing VPD, is a quick response to a phenomenon that can occur even within diurnal patterns or over longer periods, for example, the combination of heat waves and drought periods that often have major impacts on plant physiology (Grossiord et al., 2020). A mechanism of stomata regulation has been discovered involving stressor‐induced *de novo* ABA synthesis in leaves, which results in rapid stomatal closure within 15 min (McAdam & Brodribb, 2016; Sussmilch et al., 2017). High stomatal sensitivity to VPD avoids excess water loss, that is, if diurnal fluctuations of VPD are coupled with periods of reduced precipitation. Stomatal regulation and sensitivity under high VPD are noteworthy traits for conserving soil water and extending crop physiological activity as the water deficit progresses (Gholipoor et al., 2010; Schoppach & Sadok, 2013; Shekoofa et al., 2017).

Maize, *Zea mays* (L.), is one of the major global staple crops, predicted to even gain importance in the future (OECD, 2022). Further, the cultivation of maize in Central and South America has a long tradition, with Mexico being the center of domestication (Anderson, 1946; Benz, 2001). First, the wide range of represented VPD and precipitation regimes, driven by the exposition between the gulf of Mexico and the Pacific Ocean and the topography of Mexico with its low‐ and highlands, and second, the long history of maize cultivation render landraces of *Z. mays* originating from Mexico ideal to investigate the adaptation of mucilage properties and stomatal sensitivity to drought.

The majority of evaporation takes place in topsoils. Further, topsoils represent the region with the highest root length density and thereby also plant water uptake. These together render topsoils vulnerable to and most affected by drought (Lobet et al., 2014). Nodal root mucilage especially improves the rhizosphere within the topsoil. Therefore, nodal root mucilage's importance is high in reducing the severity of droughts, which occur more frequently in topsoils than in deeper soil levels under fluctuating precipitation regimes.

This study aims to (1) identify differences in nodal root mucilage traits and stomatal sensitivity crucial for drought resistance at plant‐environment boundary layers of Mexican maize landraces (in contrast to one inbred line) and (2) investigate abiotic drivers of importance for developing mucilage physical properties and stomatal sensitivity as adaptation to drought. For these objectives, we (1) grew eight maize landraces and one inbred line (B73) under controlled environmental conditions and measured for the root‐soil boundary exudation, surface tension, viscosity, and contact angle of nodal root mucilage, whereas for the leaf‐atmosphere boundary, we measured transpiration (E) and stomatal conductance (gsw), photosynthesis (A), and substomatal CO_2_ concentration (Ci) in responses to increased VPD and (2) relate measured traits to atmospheric water supply (precipitation and its seasonality) and demand (VPD) at the biogeographic origin of each landrace.

## 2 MATERIAL AND METHODS

### 2.1 Plant material and experimental setup

**FIGURE 1:**
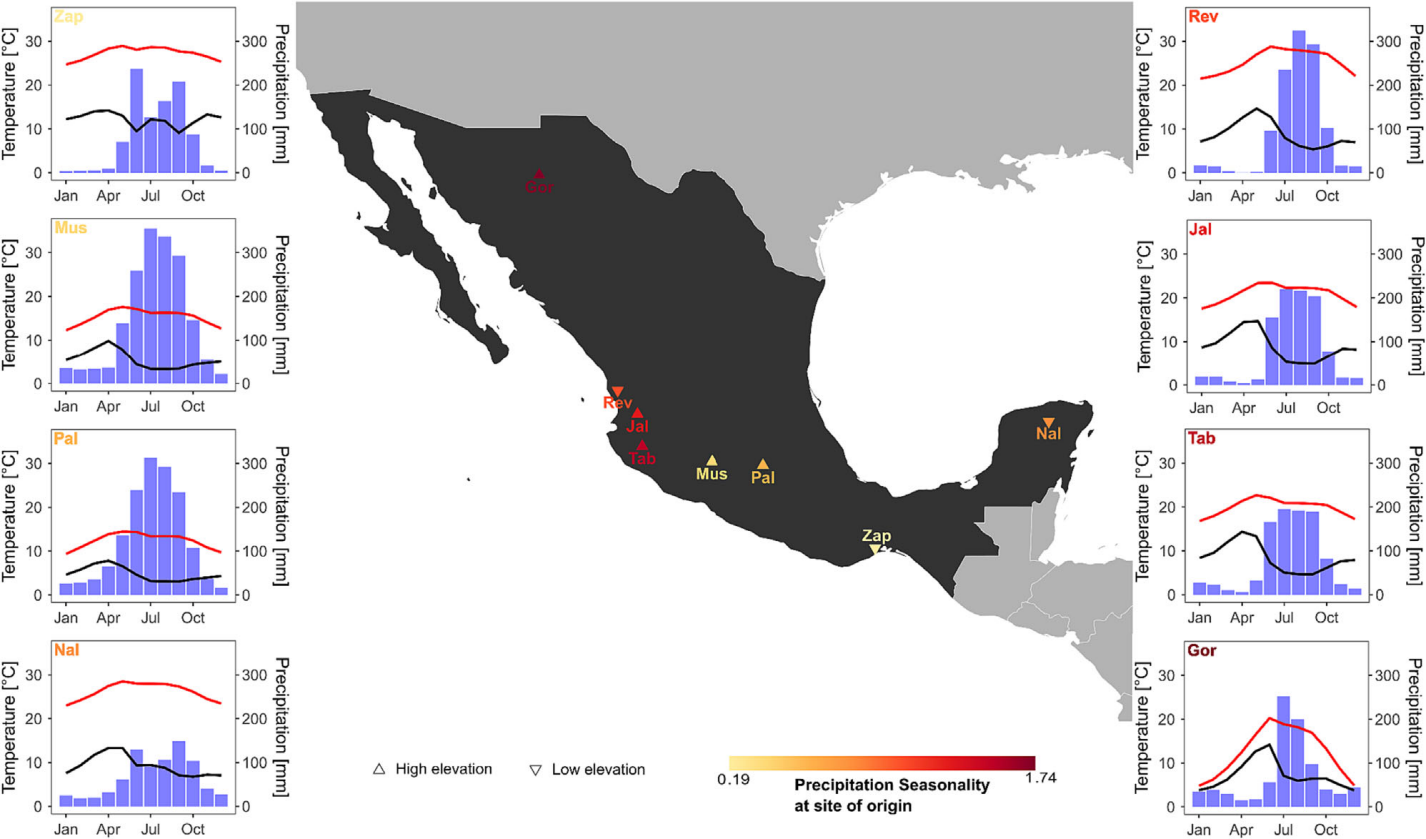
Origin of the eight Mexican landraces colored by the precipitation seasonality of the growing season (May to October). Climate diagrams of the period 1990–2020, showing monthly precipitation (bar), temperature (red line), and vapor pressure deficit (VPD, black line). Please note, scale for VPD (kPa) is temperature scale divided by 10. The tip of the triangle represents low (downwards—below 100 m a.s.l.), respectively, high (upwards—above 1000 m a.s.l.) elevation site of origin.

For this study, nine different maize varieties were used. Eight varieties were landraces originating from Mexico representing and the ninth, as reference, the inbred line B73. The landraces represent a strong climatic gradient during the rainy season from May to October, characterized by a precipitation seasonality of .19 to 1.74 and a VPD ranging between .4 and 1.1 (see Table S1 and Figure 1). To assure bio‐geographic, climatic origin, and reproducibility, all seeds were ordered via CIMMYT (www.cimmyt.org). All plants were raised under similar conditions in a greenhouse at the University of Bayreuth (49° 55′ 26.39″ N, 11° 35′ 5.39″ E). Germinated seeds were potted into 5‐L pots, with one plant per pot. The substrate of the pots was composite soil fertilized with a composite fertilizer (WUXAL SUPER 8‐8‐6) at a dosing unit of .5%. During the entire experiment, the substrate was watered as required without causing waterlogging. Pots were randomly split into three blocks, containing five individuals (*n* = 3 * 5 = 15) of each of the nine varieties.

### 2.2 Root mucilage properties

2.2.1

**Mucilage collection:** Mucilage of each plant replicate was collected from randomly selected nodal roots above ground at the beginning of tassel emergence (BBCH 51) following the method described by Ahmed et al. (2015). Briefly, the nodal roots were cut from the stem and transported to the laboratory where they have been gently cleaned from remaining soil particles. Root samples were rinsed overnight with distilled water to allow mucilage to hydrate. On the following day, excess water was discarded over a sieve with pore size 200 μm (ATECHNIK GMbH, Germany), and the hydrated mucilage was collected in a 20‐ml beacon using a syringe. The amount of fresh weight of the collected mucilage for each sample was measured using a balance (AE163 Mettler‐Toledo GMbH, Germany) and afterwards frozen at a temperature of −21°C, reflecting common procedure with root mucilage samples (see for example Nazari et al., 2020).

The root was scanned after the mucilage collection (Epson STD 4800, Japan; resolution of 400 dpi) to determine the root length and diameter using WinRhizo (Regent Instruments Inc., Canada). The root surface area was determined using the formula of the lateral surface area of a cylinder according to the method described by Nazari et al. (2020). Accordingly, the amounts of mucilage production per root surface area were determined by dividing fresh mucilage weight (mg) by total root surface area (mm^2^).

**Viscosity:** Mucilage viscosity was measured using a MCR 102 rheometer (Anthon Paar, Germany) at 20°C with a truncated cone and plate geometry (CP50‐1, *d* = 50 mm; angle of 1°); 700 μl of dissolved mucilage was gently pipetted on the bottom of a circular plate, and shear‐viscosity was measured in triplicates at shear rates ranging from .001 to 10,000 s^−1^. For comparison, the viscosity at a shear rate of .1 s^−1^ was used as it represents a shear rate realistic within the rhizosphere.

**Surface tension:** Surface tension of mucilage was determined with the pendant drop method. Mucilage was filled into a 1‐ml syringe. A drop of mucilage was suspended at the tip of the needle (8 μl with a low dispensing rate of .1 μl s^−1^). The video of the suspended drop was captured with the SCA20 software (OCA15Pro; DataPhysics, Germany). For each replicate, the surface tension was calculated and averaged from the last 10 frames of the video before the drop fell from the needle using the “pendant drop” plug‐in (Daerr & Mogne, 2016) of the Image J software (Schneider et al., 2012). Measurements were repeated 6–10 times for each mucilage sample. Viscosity and surface tension of the collected mucilage were measured at a concentration of 3 mg ml^−1^ for all genotypes.

**Contact angle:** Contact angle of mucilage was determined using the sessile drop method with a video‐based optical angle measuring device (OCA15Pro; DataPhysics; Germany). First, glass slides were cleaned in an ultrasonic bath with acetone, ethanol, and distilled water for 10 min. Dissolved mucilage was diluted to a concentration of 1.29 mg ml^−1^, and .138 ml cm^−2^ were equally dispensed on the glass slides to achieve an average mucilage cover of .138 mg cm^−2^. After drying the glass slides at ambient temperature in an exsiccator for 3–4 days, a 3‐μl drop of deionized water was placed on the dried mucilage. Shape variation of the water drop, thus of the contact angle over drop age, was recorded for 1 min using the SCA20 software (DataPhysics, Germany). For each sample, 6–10 replicates were performed. For comparison, the contact angle at a drop age of 10 s was reported in this paper.

For more details on the measurements of mucilage physical parameters, please see Knott et al. (2022).

### 2.3 Gas exchange measurements

Leaf gas exchange was measured at three consecutive days, always between 6:45 and 19:30 using a Li‐6800 (Li‐Cor, USA). One individual per maize‐variety per block was measured. One complete block per day was measured, with the blocks in chronological order from I to III. The nine measured individuals each day were assigned randomly to avoid an unintended day‐time dependent signal. In total, 27 individuals were measured, 9 varieties with each 3 individuals (*n* = 3). To ensure physiologic similarity between the measured individuals, we measured leaf gas exchange at each individual on the youngest fully developed leaf without any visual signs of damage. The cuvette (area of 6 cm^2^) was clamped after around one‐third of the leaf from the tip avoiding the midrib. To assess leaf gas exchange under atmospheric drought stress, we conducted a fully automated time series measurement with a data resolution of 1 min. The time series was 75 min long, split into 3 stepwise separated segments of VPD: (i) minute: 0–15; VPD: 1.5 kPa; pre‐stress; (ii) 16–55; 2.5 kPa; stress; (iii) 56–75; 1.5 kPa; post‐stress. With a VPD of 1.5 kPa and 2.5 kPa at 22°C temperature corresponding to a relative air humidity of 43.5% respectively 5.8%. All other environmental conditions within the leaf gas exchange chamber were controlled and held constants for all measurements at flow rate 500 μmol s⁻^1^, reference CO_2_ 485 μmol mol⁻^1^, temperature 22°C, PAR 1000 μmol m⁻^2^ s⁻^1^, 10.000 rpm ventilation.

Within each measured time series, we calculated photosynthetic performance for each of the 3 VPD‐segments at equilibrium as the mean of the last 5 min per segment. To quantify stomatal sensitivity, as the response of stomatal conductance to altered VPD, we first determined equilibration after stepwise VPD change by calculating the breakpoint of a segmented linear model using the “segmented” package (Muggeo, 2008). Next, we calculated the time (necessary to reach new equilibrium after changing VPD) and speed (slope of linear model) of equilibration as parameters to describe stomatal sensitivity to atmospheric drought stress (see Figure S1). Please note, though the Li‐6800 being very fast in changing environmental conditions (here VPD), we observed consistent “chamber‐effects” in all measured time series directly after the stepwise changes of VPD. Thus, we excluded the observations at minute 16 and 56—the very first minute after changing VPD—from every time series prior to any calculations.

### 2.4 Abiotic conditions at varieties' seed origin

For abiotic climatic conditions, we calculated monthly VPD for the period 1990–2020 based on ERA5 climate data Copernicus (Hersbach et al., 2019) and extracted precipitation seasonality (defined as $\;\Sigma \sqrt{\left(x{®}_{\text{month}}-x{®}_{\text{month total}}\right)2}$ in kg m⁻^2^ s⁻^1^) for the period 1981–2010 from NCEP‐NCAR Re‐analysis 1 (Kalnay et al., 1996). Soil moisture was extracted from “Global distribution of plant‐extractable water capacity of soil” (Dunne & Willmott, 1996). These climatic parameters have been chosen, as they represent atmospheric water demand, respectively, supply and link atmospheric and edaphic plant water availability (see also Nazari et al., 2020).

### 2.5 Analysis

To test for differences of mucilage properties between varieties, we conducted an analysis of variance (ANOVA) (linear model: response ~ variety) followed by a post hoc test for pairwise comparisons (Sidak). Differences in photosynthesis response to atmospheric drought stress (VPD step) were tested using an ANOVA (linear model: response ~ variety * VPD‐step) followed by a post hoc test to either compare within a variety between different VPD steps or between varieties within the same VPD step. All model assumptions (normal distribution and homogeneity of variances) were estimated based on standard graphs (histogram, qqplot). Correlation analysis was conducted (a) between mucilage and photosynthesis properties and (b) between the abiotic conditions at the varieties' seed origin and the mucilage and photosynthesis properties to test if and which abiotic environmental conditions related to soil water availability and atmospheric water demand shape trait development. All statistical analyses were performed using R version 4.1.0 (R Core Team, 2022).

## 3 RESULTS

### 3.1 Mucilage properties

**FIGURE 2:**
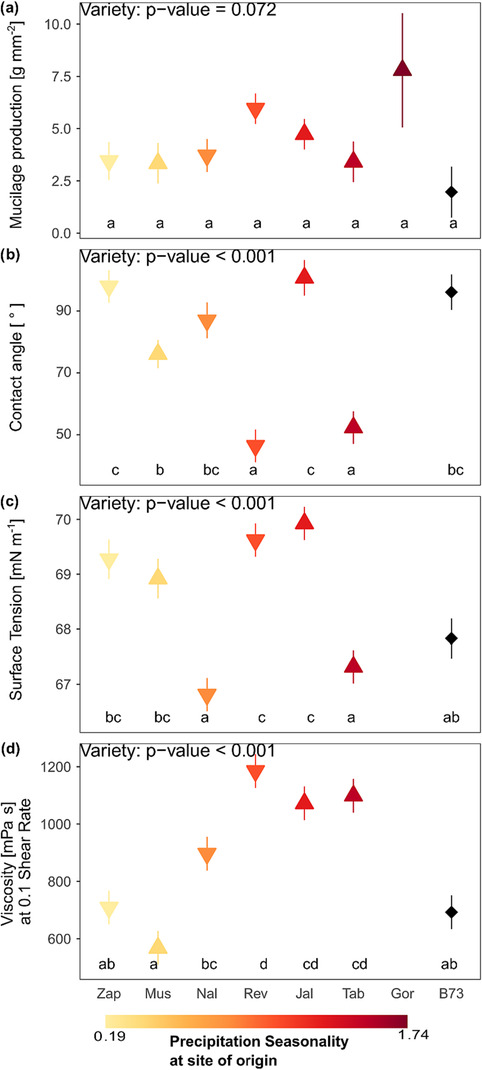
Mucilage properties of different varieties showing (a) mucilage production, (b) contact angle after 10 s, (c) surface tension, and (d) viscosity at shear rate .1 s^−1^. Displayed are mean and standard error of linear model, with the respective model effect of variety displayed in the top left corner. Letters indicate significant pairwise differences based on post hoc test. Precipitation seasonality at site of varieties' origin is indicated by color ramp, with black representing the inbred line B73 and the tip of the triangle representing low (downwards–below 100 m a.s.l.), respectively, high (upwards—above 1000 m a.s.l.) elevation site of origin.

Mucilage production per unit of root surface area did not differ significantly among varieties (effect of variety on mucilage production: *p* = .072, *F* = 1.98). All landraces revealed a higher mucilage production than the inbred line B73 with a mean of 1.97 g mm^−2^ (SE: .66 g mm^−2^, SD: 1.49 g mm^−2^). Mucilage production within the landraces ranges twofold from a mean of 3.34 g mm^−2^ (SE: .75 g mm^−2^, SD: 2.13 g mm^−2^) of the low precipitation seasonality (.27) variety Mus to 7.79 g mm^−2^ measured for the highest precipitation seasonality (1.74) variety Gor, though the latter has no replicates as not enough nodal roots developed to collect sufficient mucilage. The second highest mucilage production was obtained from variety Rev (intermediate precipitation seasonality .519) with a mean of 5.95 g mm^−2^ (SE: 1.22 g mm^−2^, SD: 4.58 g mm^−2^) (Figure 2a).

The contact angle of dried mucilage determines its water repellency. If the contact angle is less than 90°, mucilage remains hydrophilic, whereas if the contact angle is larger than 90°, the mucilage exhibits water repellency upon drying. Here, the contact angle of the landraces ranged between 46.4° (SE: 1.89°, SD: 4.62°) and 101.0° (SE: 8.66°, SD: 19.4°). B73, the inbred line, with a mean contact angle of 96.1° (SD: 14.4°, SE: 6.45°) is not significantly different from four of the six analyzed landraces here and shows a slight water repellency. Mucilage of the landraces Rev (mean: 46.4°, SD: 4.62°, SE: 1.89 °) and Tab (mean: 52.3°, SD: 15.6°, SE: 6.37°) is wettable with contact angles significantly lower than the remaining maize varieties ranging between a mean of 76.1° and 101° (effect of variety on contact angle: *p* < .001, *F* = 16.98, Figure 2b). Important to mention is that two landraces with comparably high VPD (Zap VPD: 1.218; Jal VPD: .887) but contrasting low (.188) and intermediate (.519) precipitation seasonality as well as the inbred line exhibited contact angles above 90°, thereby indicating water repellency upon drying of the mucilage.

Surface tension of maize mucilage is lower than surface tension of water (72.53 mN m^−1^ at 20°C) and ranges between 66.8 mN m^−1^ (SD: 2.27 mN m^−1^, SE: .717 mN m^−1^) and 69.9 mN m^−1^ (SD: .0750 mN m^−1^, SE: .0237 mN m^−1^) for the landraces with the tested inbred line B73 being intermediate at 67.8 mN m^−1^ (SD: .627 mN m^−1^, SE: .237 mN m^−1^). Four of the landraces reveal a higher surface tension than the landrace Nal and the landrace Tab (effect of variety on surface tension: *p* < .001, *F* = 15.26, Figure 2c). A low surface tension, that is, lower than the surface tension of water, enhances and prolongs root‐water uptake.

The viscosity at a shear rate of .1 s^−1^ of the landraces ranged between 568.2 mPa·s (SD: 87.28 mPa·s, SE: 50.39 mPa·s) and 1185.2 mPa·s (SD: 35 mPa·s, SE: 20.21 mPa·s) with the inbred line at the lower limit with 692.53 mPa·s (SD: 213.02 mPa·s, SE: 122.99 mPa·s). The effect of variety on viscosity is significant (*p* < .05, *F* = 16.36). Three varieties (Rev, Jal, Tab) originating from places characterized by intermediate precipitation seasonality (.519–.533) and comparable high VPD (.841–.887 kPa) show a higher viscosity as the inbred line B73 (Figure 2d). The changes of viscosity with increasing shear rate (flow curves) for the investigated landraces are shown in Figure S2.

### 3.2 Gas exchange

**FIGURE 3:**
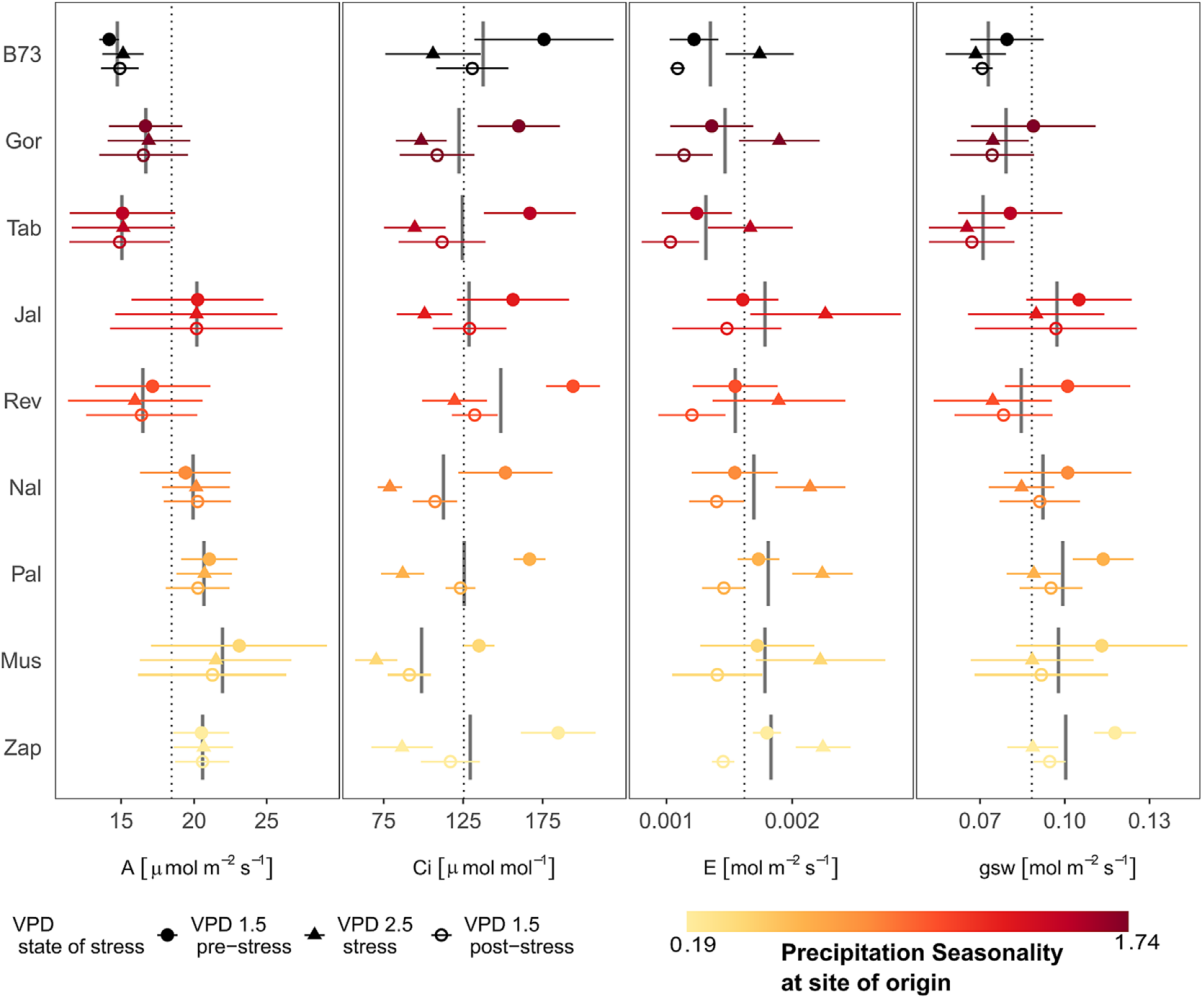
Leaf‐level gas exchange of different varieties expressed as net assimilation (A), intercellular CO_2_ concentration (Ci), transpiration (E), and stomatal conductance (gsw) pre‐, during, and post‐stress as filled blue circles and filled red triangles, respectively, open blue circles. Shown are mean and standard errors, dashed lines indicate overall mean of all varieties, and gray bars indicate overall mean of each single variety. Precipitation seasonality at site of varieties' origin is indicated by color, with black representing the inbred line B73.

All leaf‐level gas exchange parameters are affected by maize variety. Net assimilation (A) of the tested maize varieties is not affected by atmospheric drought stress, though the varieties reveal different net assimilation rates (*p*_VPD_ = .977; *p*_variety_ < .01). Under increased atmospheric drought stress (increased VPD) transpiration (E, *p*_VPD_ < .001; *p*_variety_ < .05) increases, while stomatal conductance (gsw, p_VPD_ < .01; *p*_variety_ < .01) and intercellular CO_2_ concentration (Ci, *p*_VPD_ < .001; *p*_variety_ < .001) decreases (Figure 3).

After release of atmospheric drought stress, stomatal conductance remains at the low levels during stress, though none of the visible changes due to altered VPD is statistically significant. Transpiration post‐stress is lower than during stress for all varieties besides Rev and Tab, which represent varieties with comparable precipitation seasonality and VPD (*p*_stress:post‐stress_ < .05 for all varieties). Yet, transpiration visually increased from pre‐stress to stress conditions, and this trend is not significant. Intercellular CO_2_‐concentration is reduced during as well as post‐stress in comparison with pre‐stress levels for all varieties excluding Jal where post‐stress is similar to pre‐stress levels (Figure 3).

**FIGURE 4:**
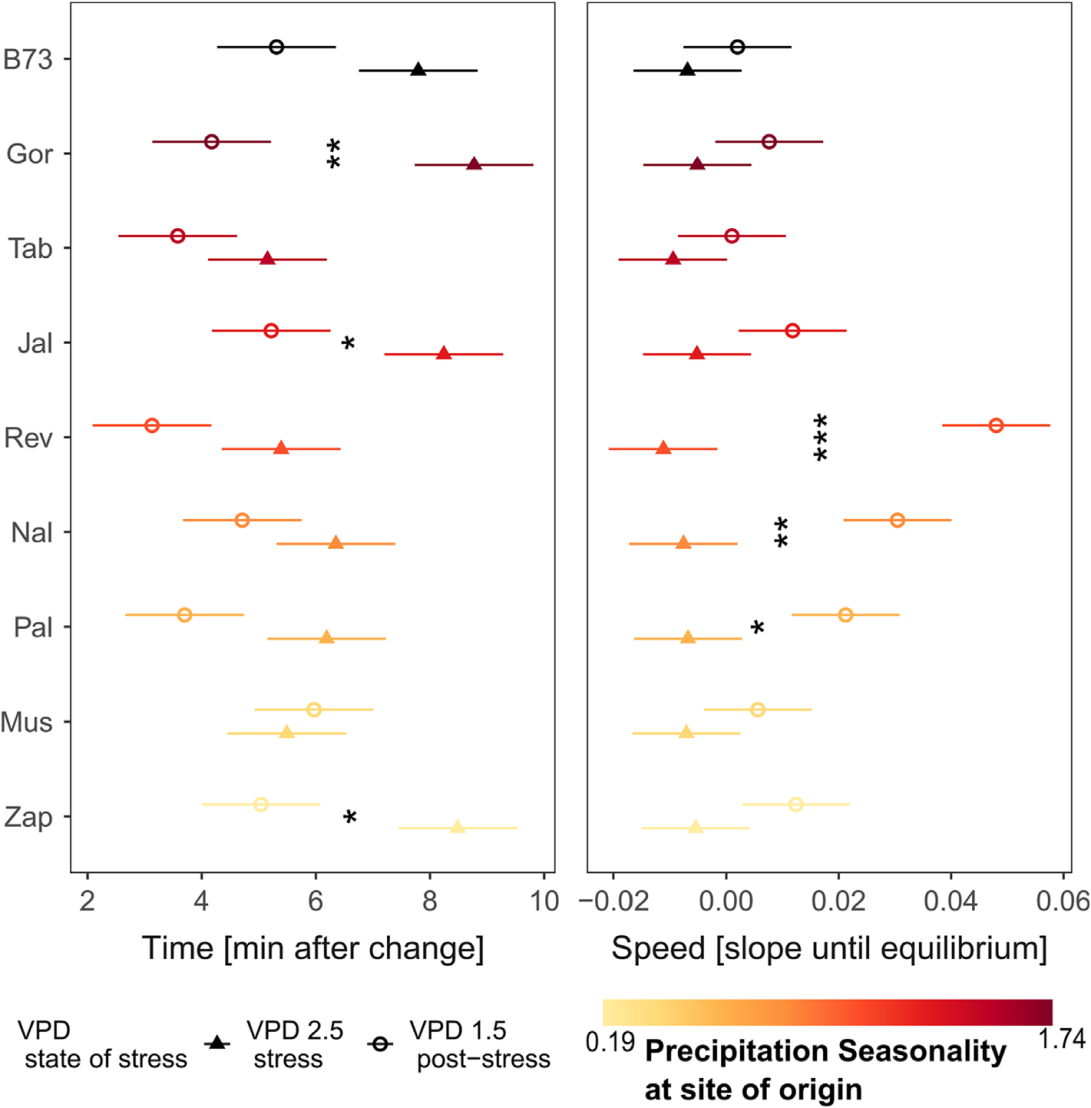
Time (left column) and speed (right column) of stomatal conductance recovery after reaching (filled triangles) or being released (open circles) from atmospheric VPD stress for different varieties. Shown are mean and standard errors of linear model. Asterisks indicate significant differences between the response to or the release from stress. Precipitation seasonality at site of varieties' origin is indicated by color, with black representing the inbred line B73.

In general, time needed to reach a novel equilibrium is longer if leaves are exposed to atmospheric drought stress, then it is if they are released from atmospheric drought stress. Similarly, the speed of recovery after stress release is higher than after stress induction. Both, time needed to equilibrate and speed of equilibration to and after stress of stomatal conductance, differ within varieties (Figure 4). Though, time and speed of stomatal recovery did not differ between varieties, except the speed of post‐stress recovery of variety Rev is higher (slower) than of variety Tab (*p* < .05) and the inbred line B73 (*p* < .05) (Figure 4 right panel).

### 3.3 Relation between mucilage, stomatal sensitivity, and environment at the origin

Mucilage production significantly correlates with precipitation seasonality (*R* ^2^ = .88, *p* < .01), and viscosity significantly correlates with precipitation seasonality (*R* ^2^ = .93, *p* < .01) and soil moisture (*R* ^2^ = .91, *p* < .05), indicating the potential importance of adapting mucilage properties to precipitation regimes. Further, mucilage contact angle correlates with various stomatal responses to atmospheric drought stress, and viscosity correlates with time needed for equilibration to novel conditions (Table 1). Contrary to the correlation between abiotic drivers influencing plant soil water availability and root mucilage properties, none of the here investigated abiotic environmental predictors (neither edaphic nor climatic) significantly correlates with the stomatal response to atmospheric drought stress of the investigated maize varieties.

**TABLE 1:** Direction (+/−) and strength (*R* ^2^) or Pearson's correlations of root and leaf‐level gas exchange parameters. All correlations are based on the means per variety due to differing sampling extent. Shown are only significant correlations with indicated *p* value.

Mucilage property	Environmental property	*R* ^2^	*p* value
Production	Seasonality of precipitation	(+) .88	<.01
Viscosity	Seasonality of precipitation	(+) .93	<.01
Viscosity	Soil moisture	(−) .91	<.05

Mucilage property	Stomatal sensitivity		
Contact angle	Stomatal conductance | stress	(+) .87	<.05
Contact angle	Stomatal conductance | post‐stress	(+) .89	<.05
Contact angle	Speed to equilibrium | stress	(+) .96	<.01
Contact angle	Time to equilibrium | stress	(+) .87	<.05
Viscosity	Time to equilibrium | post‐stress	(−) .82	<.05

## 4 DISCUSSION

For the first time, we showed that maize plants may have adapted the physical properties of their exuded mucilage to adapt to environmental conditions. Indeed, mucilage properties correlated with environmental conditions influencing plant water availability (i.e., precipitation seasonality and soil moisture availability). Yet, no correlation was detected between stomatal regulation and environmental conditions at the origin of landraces. Further, we detected a strong correlation between contact angle of mucilage and stomatal sensitivity to drought. Besides contact angle, we found little evidence for a correlation between mucilage properties and stomatal‐associated traits on the whole. These results suggest, first, a separate development of resistance to on the one hand atmospheric and the other hand edaphic drought. Precisely, the reversible, short‐term reaction to atmospheric drought (stomatal sensitivity) seems not to be strongly linked to VPD at the site of origin, whereas the permanent, long‐term adaptation to edaphic drought (mucilage properties) revealed linkage to plant water availability at the site of origin. Second, the link between stomatal sensitivity and mucilage contact angle hint towards stomatal regulation preventing excess transpirational water loss to avoid mucilage drying, thereby reducing the maladaptive characteristics of dry mucilage and likely delay the occurrence of critical levels of xylem dysfunction. These results suggest landraces grown under highly variable climates may incorporate pre‐adaptations to edaphic drought.

### 4.1 Mucilage properties

Because root mucilage improves root soil water access and rhizosphere conductivity (Ahmed et al., 2014; Carminati et al., 2016; Naveed et al., 2019), mucilage exudation bears one potential strategy to maintain high transpiration demand and carbon assimilation during soil drying. However, mucilage can become hydrophobic upon drying, which causes water repellent behavior in the rhizosphere (Ahmed et al., 2016; Kaltenbach et al., 2018) and increases the rewetting time of soil (Zarebanadkouki & Carminati, 2014). The hydrophobic nature of dry mucilage exhibited by two landraces and the inbred line might not only have negative effects on plant water uptake. While sufficient and hydrated mucilage may facilitate the water uptake of young roots, hydrophobic mucilage may insulate old roots, with decreased water uptake capacity, and thereby increase water availability for young roots. Therefore, by controlling amounts and location of mucilage exudation, plants can control which parts of the root have improved access and which are isolated from soil water (Carminati & Vetterlein, 2013). Water uptake and its radial transport is highest in young roots, which also represent the primary source of mucilage exudation. Whereas in older roots, axial transport of water dominates the roots function (Frensch & Steudle, 1989; Lobet et al., 2014), which might favor the bimodal function of mucilage to improve (at young) or impede (at old) root water uptake. As mucilage here was collected at nodal roots from aboveground, we are not able to draw inference depending on root age. Yet, we discuss mucilage properties in the light of increased and elongated hydraulic connectivity in the rhizosphere under soil drying, as this represents one of root mucilages primary functions (Carminati et al., 2010, 2011) and likely is the most important function of nodal root mucilage due to the drought vulnerable nature of topsoils (Lobet et al., 2014).

The comparably high amounts of mucilage production from Mexican landraces with extensive aerial root development measured in this study are in line with previous findings presented by Van Deynze et al. (2018). Although mucilage production did not differ significantly between tested maize varieties, all landrace varieties had higher mucilage production than the inbred line B73. This may result from past breeding efforts to increase crop yield (Voss‐Fels et al., 2019), which not only, for instance, reduced pathogen resistance (Wulff & Dhugga, 2018) but also likely affected belowground root traits and their exudates. For example, root system biomass has decreased over time with cultivar development and has smaller, steeper, and deeper roots (Aziz et al., 2017; Friedli et al., 2019; Ren et al., 2022). Therefore, the net mucilage exudation of the inbred line may be minimal in comparison with landrace varieties.

Landraces investigated here revealed contact angles smaller or larger than 90°, with the inbred line (B73) exhibited contact angles above 90°, indicating water repellency upon drying of the mucilage. These high contact angles, causing a hydrophobic surface of mucilage, can be caused by differences in mucilage chemical composition, the amphiphilic nature of mucilage composing polymers and the amount of cations (Knott et al., 2022). Mucilage with high contact angles often contains more phospholipids than those with a lower contact angle. The slow rewetting of the rhizosphere after drying of varieties with contact angles above 90° may locally limit the water uptake of the roots during drying and re‐wetting cycles (Zarebanadkouki & Carminati, 2014). The effect of drying and wetting cycles in the rhizosphere mostly depends on the soil types, and as Benard et al. (2018) demonstrated, such effect is more relevant in sandy soil, because of its smaller specific surface area. Therefore, extended soil drying may cause the mucilage to become water repellent in the rhizosphere (Moradi et al., 2012), may reduce plant water uptake even after the soil is re‐wetted (Zarebanadkouki et al., 2016), and consequently could delay the recovery of the leaf water potential and stomatal conductance.

The detected differences between the inbred line B73 and the eight Mexican landraces might result from long‐term crop breeding and cultivation. We thoroughly want to note that generalization of differences between inbred lines and landraces cannot be drawn from this study, as we only used one inbred line for comparison. Yet, the selection of traits to increase yield and reduce generation time of agricultural used breeds over the past centuries constrains the development of resistances (Milla et al., 2015). These by past breeding induced shifts towards resource‐acquisitive traits are especially effective under favorable conditions, yet cause drops in crop morpho‐physiological and, ultimately, agro‐economic performance under stress (McCoy et al., 2022; Milla et al., 2015). As this reduction of resistances and the shift towards resource‐acquisitive plant traits is a general trend in past crop‐breeding, we would expect to detect similar differences between landraces and inbred lines in further studies.

While contact angle is affecting mucilage behavior upon drying, mucilage's viscosity affects the speed of the drying process (Brinker & Scherer, 2013; Kroener et al., 2014). The viscosity of mucilage determines its ability to spread within the pore space of the rhizosphere and the ability of water to be transported within the mucilage (Knott et al., 2022). The exudation of mucilage with high viscosity and low surface tension is one of the mechanisms maintaining the connectivity of the liquid phase in the rhizosphere during soil drying (Benard et al., 2019; Carminati et al., 2017), which is of special importance during the early life stages and a “young” rhizosphere (Carminati & Vetterlein, 2013). Although we did not detect significant differences between the varieties' viscosity, the differences measured between the varieties span a 2.5‐fold increase, which likely affects rhizosphere hydraulic connectivity during drying and re‐wetting cycles. The previously discussed differences in mucilage physical properties with regard to contact angle, viscosity, and surface tension are affected by soil chemical characteristics, for example, pH (Knott et al., 2022); thus, the differences in mucilage properties reported here might change if the varieties are grown under varying substrate properties or in soil local at their respective site of origin. Nonetheless, as all varieties investigated here have been grown in the same, standardized substrate, we argue the differences between varieties mucilage properties to be representative for the given substrate.

The variation in exuded mucilage's properties by maize landraces suggests that mucilage properties are partly governed by genotypes and may be shaped by the variability in plant available water during the rainy season, the season of cultivation. Thereby, mucilage properties may entail an adaptation to enhance and prolong plant water uptake (Carminati & Vetterlein, 2013) under future climatic scenarios (Satoh et al., 2022). Increasing amounts of produced mucilage as well as higher viscosity of mucilage with increasing precipitation seasonality enhance rhizosphere conductivity under frequent drying and rewetting cycles (Ahmed, Zarebanadkouki, et al., 2018; Kroener et al., 2018). Mucilage concentration in the rhizosphere is increasing with the amount of produced mucilage. If mucilage concentration is high within the rhizosphere, the saturated rhizosphere hydraulic conductivity is independent of soil particle size. Thus, mucilage is increasing the water content especially if under tension due to drying (Kroener et al., 2018). Hydraulic conductivity in the rhizosphere will decrease if mucilage viscosity is increasing; thereby, a higher viscosity of mucilage imposes light stress in plants and decreases transpiration. The reduction in water consumption, caused by decreased transpiration, allows the plant to withstand prolonged periods without precipitation (Ahmed, Zarebanadkouki, et al., 2018). This counterintuitive behavior of increasing drought resistance by reducing root‐water uptake arises from short‐term mechanisms coupling stomatal conductance, plant water potential, and root hydraulic conductance and is explained in detail by Tardieu and Parent (2017).

### 4.2 Gas exchange

Despite the reduction of stomatal conductance and substomatal CO_2_ concentration under elevated atmospheric drought, the net assimilation (A) was unaffected by increasing VPD. The lack in response of net assimilation (A) to increased VPD is caused by the C4‐type photosynthesis of maize. C4‐photosynthesis is characterized by an energy‐driven CO_2_‐fixation (Osmond et al., 1982), maximizing assimilation rates at lower stomatal conductance and lower substomatal‐ambient CO_2_ ratios (Ci/Ca) in comparison with C3‐photosynthesis (Pinto et al., 2014). Another underlying mechanism might be the increase in photosynthetic efficiency caused by high leaf nitrogen concentrations (López et al., 2021); unfortunately, we are unable to test this. The reduction of stomatal conductance (gsw), substomatal CO_2_‐concentration (Ci), and transpiration (E) under elevated VPD was expected and shown manifold before (e.g., Oren et al., 1999). With the short‐term (20 min) exposure of a given leaf area to high VPD, we were able to investigate the reaction and its speed of leaf gas exchange to stress implementation and stress release. Though, this stress‐induced reaction likely differs from the reaction of entire individuals or stands exposed to high VPD, especially if exposure is long term. Interestingly, the post‐stress recovery of gsw, Ci, and E did not reach the pre‐stress level, indicating a rather conservative stomatal behavior after abrupt stress release. It was shown that *de novo* synthesis of ABA in response to increased VPD can occur within 20 min (McAdam & Brodribb, 2015, 2016). The little time (ranging from around 4 to 9 min) needed to reach their respective new equilibrium under stress conditions of the here investigated varieties might add further evidence to the rapid *de novo* synthesis of ABA. Post‐stress ABA concentrations remain high caused by a delayed ABA catabolism and a NCED‐transcript levels (McAdam & Brodribb, 2016), ultimately resulting in the here observed hysteresis—the “not‐recovery” after stress release, respectively, the different time needed and speed to equilibrate to stress or post‐stress.

### 4.3 Relation between mucilage, stomatal sensitivity, and environment at the origin

Our results suggest that mucilage properties and stomatal sensitivity to VPD may represent adaptations to environmental processes that differ fundamentally in their nature. Atmospheric drought, by an increase in VPD, is a pattern that can occur relatively fast (Grossiord et al., 2020). While on the other hand edaphic drought is a pattern that emerges over longer time frames. Similarly, the adaptation to atmospheric drought—stomatal sensitivity—is a fast, reversible process; on the other hand, mucilage properties are properties expressed over an individual's entire life stage as its function is manifold, for example, for belowground biotic and abiotic interactions (el Zahar Haichar et al., 2014). Although mucilage properties and stomatal sensitivity represent adaptations to two different environmental phenomena, we found a relationship between contact angle, viscosity—both of them important properties for mucilage's behavior during drying and rewetting cycles (Ahmed et al., 2016; Kaltenbach et al., 2018; Knott et al., 2022)—and stomatal sensitivity to increased VPD. As stomatal regulation controls the current transpirational water loss, the plant indirectly affects the speed of soil drying and ultimately delays the drying out of mucilage. By this, we argue, stomatal sensitivity may reduce the plants risk to suffer from the maladaptive effects of dry mucilage, in particular its water repellency upon drying.

In a similar vein, the increasing tension on the plant internal water column induced by decreased soil water supply, increased atmospheric water demand, or the combination of both (Dixon & Joly, 1895) ultimately causes hydraulic dysfunction by cavitation and spreading embolism within the xylem (Sperry, 2000; Tyree & Sperry, 1989). It was shown that plants close their stomata early to prevent critical levels of xylem dysfunction (Creek et al., 2020). Stomatal closure is regulated biochemical and biophysical. Differences in osmotic potential and turgor pressure between guard cells and epidermal cells biophysically regulate stomatal opening (for a review, see Buckley, 2005 and references in there). Biochemical regulation of stomatal opening is to major portions driven by leave ABA concentrations. Diurnal fluctuations of VPD and the according stomatal response are key in regulating diurnal changes in land plant gas exchange (Zhao & Running, 2010). With increasing VPD, the rate‐limiting gene NCED3 is upregulated, causing an increase in leave ABA concentration and ultimately a change in guard cell turgor pressure via ABA activated ion channels (Sussmilch et al., 2017). Though, the accumulation of root born ABA inducing rapid stomatal closure in response to increased VPD was rejected (Buckley, 2016), and the accumulation of root‐born ABA can cause constant closure of stomata in response to dry soils (Comstock, 2002; Davies & Zhang, 1991). Thereby, leave ABA concentration reflects the sum of, first, the short‐term response to rapid changes in VPD, driven by *de novo* synthesis of ABA within the leaves (Buckley, 2016), and second, the accumulation of root born ABA in response to sustained soil drying (Comstock, 2002; Davies & Zhang, 1991). Root mucilage enhances rhizosphere conductivity and connectivity, thus plant water availability. This by mucilage improved soil water availability likely delays the accumulation of root born ABA, allowing for elongated carbon assimilation under fluctuating precipitation regimes. Thus, the interplay between a high stomatal sensitivity, to reduce excess transpirational water loss and thereby fast soil drying in periods of high atmospheric water demand, and the physical properties of exudated root mucilage, to enhance plant water availability by improving rhizosphere connectivity and conductivity under drying soils, may reduce the risk of critical xylem dysfunction in plants.

### 4.4 Conclusion

The identification of landraces with pre‐adapted trait sets with regard to drought resistance is of utmost importance, for example, trait combinations such as high viscosity, low surface tension, and low contact angle as exhibited in one of the here investigated landraces (Tab). The results presented here hint towards first indications of a strong environmental selective force of seasonality in plant water availability on mucilage properties as well as regulatory stomatal effects to avoid mucilage's maladaptive potential upon drying and delay critical levels of xylem dysfunction upon soil drying. By this, landraces from highly seasonal climates may exhibit beneficial mucilage and stomatal traits to prolong plant functioning under edaphic drought and thereby offer a promising opportunity to further study the coordination between mucilage and stomatal properties and environmental factors shaping their development.

## AUTHOR CONTRIBUTIONS

All authors contributed to and approved the final version of the manuscript. AA, BJB, MAA, and AS designed the study. AA and BJB conducted the experiment. AA, DD, MK, and M‐PW conducted the measurements on root mucilage properties. RJHS and MAA provided seed material.

## CONFLICT OF INTEREST STATEMENT

No conflict of interest is declared.

## Supporting information


**Figure S1:** Scheme to visualize the here chosen quantification of stomatal sensitivity quantified within the three‐step VPD time series conducted. Blackline represents a characteristic behavior of stomatal conductance. Breakpoint for new equilibrium was calculated using a broken stick linear model. Time to reach equilibrium: T_Breakpoint_ ‐ T_VPD‐change_. Speed to reach equilibrium: slope of lm (stomatal conductance ~ time) for stomatal conductance between [T_Breakpoint_ ‐ T_VPD‐change_].

**Figure S2:** Reduction of mucilage viscosity with increasing shear rate for the investigated landraces. The origin of landraces is indicated by low (long dash) or high (solid) elevation, with the colors indicating the precipitation seasonality at the site of origin ranging from low (.19 ‐ light yellow) to high (1.74 ‐ dark red). Gray horizontal lines indicate the shear rates .1 and 1, which represent a common range of shear rates occurring within the rhizosphere.

**Table S1:** Information on the used landraces, including CIMMYT code, geographic coordinates, elevation and precipitation seasonality and VPD of the rainy season (May–October) at the site of origin

## Data Availability

The data that support the findings of this study are openly available in Zenodo at 10.5281/zenodo.8214731.
