# Supplementary figures and images for: Differences in mucilage properties and stomatal sensitivity of locally adapted Zea mays in relation with precipitation seasonality and vapour pressure deficit regime of their native environment
Source: Plant Direct. 2023 Aug 17;7(8):e519. doi: 10.1002/pld3.519 (PMC10435965; doi:10.1002/pld3.519)

Stomatal conductance

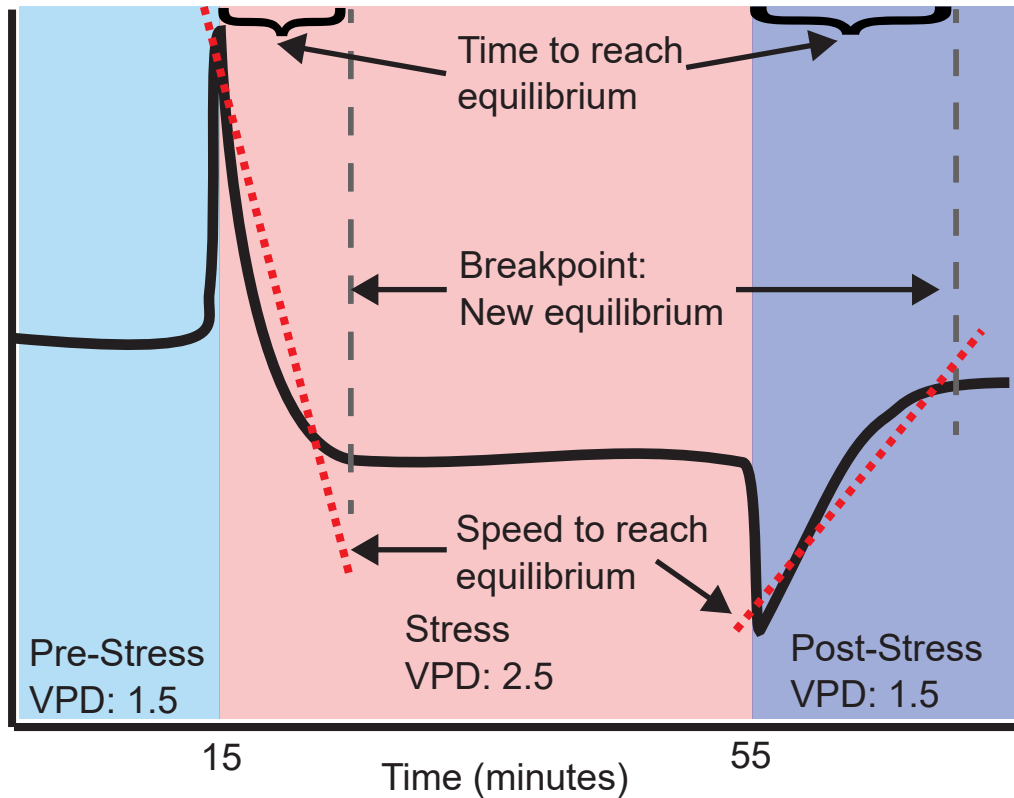

Supplement: Supplementary file 1 — Figure S1: Scheme to visualize the here chosen quantification of stomatal sensitivity quantified within the three‐step VPD time series conducted. Blackline represents a characteristic behavior of stomatal conductance. Breakpoint for new equilibrium was calculated using a broken stick linear model. Time to reach equilibrium: TBreakpoint ‐ TVPD‐change. Speed to reach equilibrium: slope of lm (stomatal conductance ~ time) for stomatal conductance between [TBreakpoint ‐ TVPD‐change]. [file PLD3-7-e519-s001.pdf]

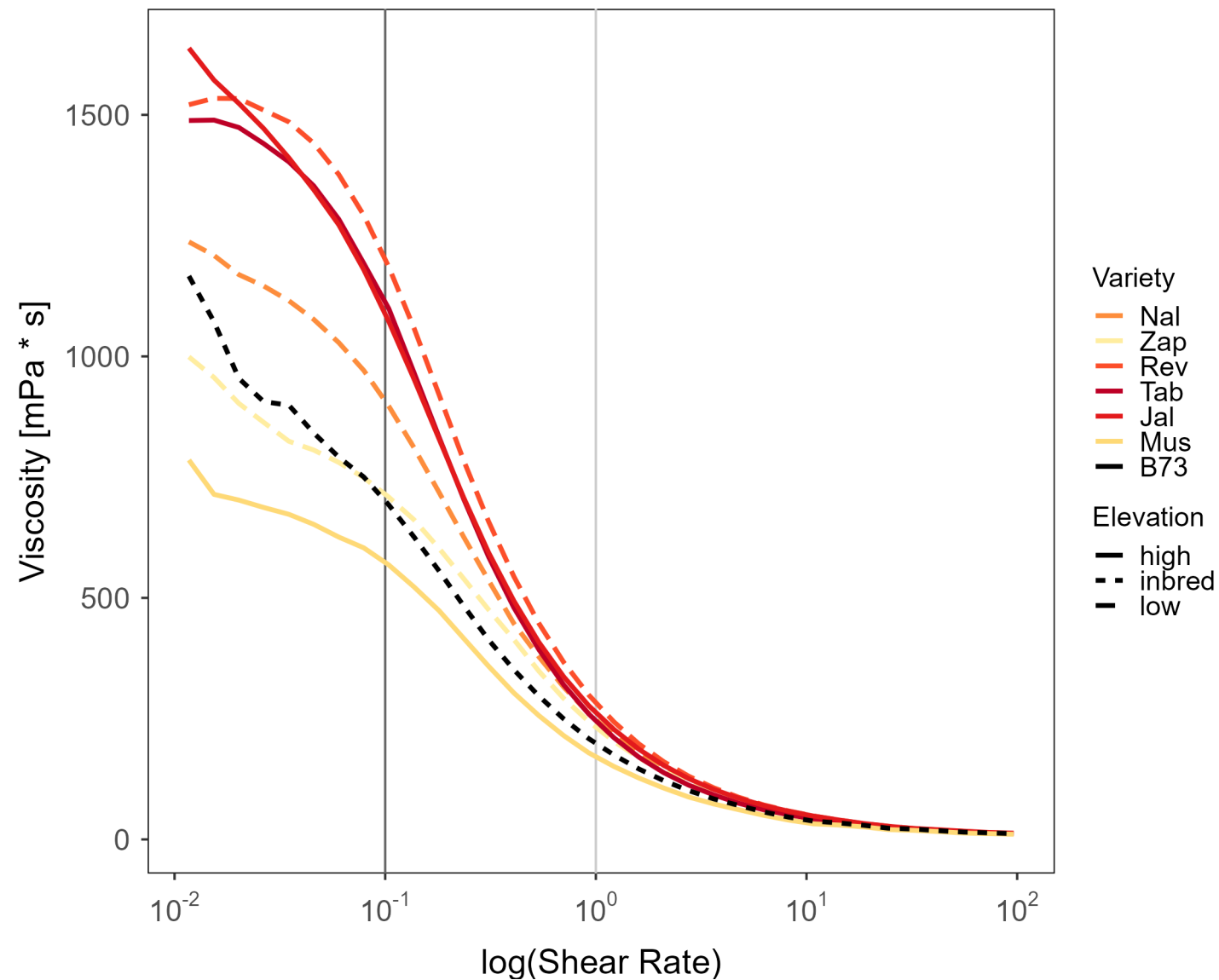

Supplement: Supplementary file 2 — Figure S2: Reduction of mucilage viscosity with increasing shear rate for the investigated landraces. The origin of landraces is indicated by low (long dash) or high (solid) elevation, with the colors indicating the precipitation seasonality at the site of origin ranging from low (.19 ‐ light yellow) to high (1.74 ‐ dark red). Gray horizontal lines indicate the shear rates .1 and 1, which represent a common range of shear rates occurring within the rhizosphere. [file PLD3-7-e519-s003.pdf]
