# Supplementary material for: Differences in mucilage properties and stomatal sensitivity of locally adapted Zea mays in relation with precipitation seasonality and vapour pressure deficit regime of their native environment
Source: Plant Direct. 2023 Aug 17;7(8):e519. doi: 10.1002/pld3.519 (PMC10435965; doi:10.1002/pld3.519)
Supplement: Supplementary file 3 — Table S1: Information on the used landraces, including CIMMYT code, geographic coordinates, elevation and precipitation seasonality and VPD of the rainy season (May–October) at the site of origin [file PLD3-7-e519-s002.docx]

Supporting Material

*Table S1: Information on the used landraces, including CIMMYT code, geographic coordinates, elevation and precipitation seasonality and VPD of the rainy season (May-October) at the site of origin*

| **Code** | **Type** | **CIMMYT** | **Latitude** | **Longitude** | **Precipitation seasonality** | **VPD** | **Elevation** |
| --- | --- | --- | --- | --- | --- | --- | --- |
| Zap | Landrace | OAXA521 | 16.35 | -95.23 | 0.188 | 1.116 | 44 |
| Mus | Landrace | MICH320 | 19.42 | -101.56 | 0.266 | 0.443 | 2483 |
| Pal | Landrace | MEXI5 | 19.29 | -99.57 | 0.268 | 0.401 | 2597 |
| Nal | Landrace | YUCA7 | 20.85 | -88.52 | 0.378 | 0.912 | 31 |
| Rev | Landrace | NAYA15 | 21.95 | -105.22 | 0.519 | 0.882 | 13 |
| Jal | Landrace | NAYA6 | 21.11 | -104.45 | 0.519 | 0.750 | 1099 |
| Tab | Landrace | JALI43 | 19.97 | -104.27 | 0.533 | 0.684 | 1355 |
| Gor | Landrace | CHIH140 | 29.65 | -108.25 | 1.74 | 0.879 | 2141 |
| *B73* | *Inbred* | *RS20-3487xSIB* |  |  |  |  |  |
